# Adverse Pathology after Radical Prostatectomy of Patients Eligible for Active Surveillance—A Summary 7 Years after Introducing mpMRI-Guided Biopsy in a Real-World Setting

**DOI:** 10.3390/bioengineering10020247

**Published:** 2023-02-13

**Authors:** Benedikt Ebner, Maria Apfelbeck, Nikolaos Pyrgidis, Tobias Nellessen, Stephan Ledderose, Paulo Leonardo Pfitzinger, Yannic Volz, Elena Berg, Benazir Enzinger, Severin Rodler, Michael Atzler, Troya Ivanova, Dirk-André Clevert, Christian Georg Stief, Michael Chaloupka

**Affiliations:** 1 Department of Urology, Ludwig-Maximilians-University of Munich, 81377 Munich, Germany; 2 Department of Pathology, Ludwig-Maximilians-University of Munich, 81377 Munich, Germany; 3 Department of Radiology, Interdisciplinary Ultrasound Center, Ludwig-Maximilians-University of Munich, 81377 Munich, Germany

**Keywords:** active surveillance, mpMRI fusion biopsy, upgrading, prostate cancer

## Abstract

Objective: Over the last decade, active surveillance (AS) of low-risk prostate cancer has been increasing. The mpMRI fusion-guided biopsy of the prostate (FBx) is considered to be the gold standard in preoperative risk stratification. However, the role of FBx remains unclear in terms of risk stratification of low-risk prostate cancer outside high-volume centers. The aim of this study was to evaluate adverse pathology after radical prostatectomy (RP) in a real-world setting, focusing on patients diagnosed with Gleason score (GS) 6 prostate cancer (PCa) and eligible for AS by FBx. Subjects and Methods: Between March 2015 and March 2022, 1297 patients underwent FBx at the Department of Urology, Ludwig-Maximilians-University of Munich, Germany. MpMRI for FBx was performed by 111 different radiology centers. FBx was performed by 14 urologists from our department with different levels of experience. In total, 997/1297 (77%) patients were diagnosed with prostate cancer; 492/997 (49%) of these patients decided to undergo RP in our clinic and were retrospectively included. Univariate and multivariable logistic regression analyses were performed to evaluate clinical and histopathological parameters associated with adverse pathology comparing FBx and RP specimens. To compare FBx and systematic randomized biopsies performed in our clinic before introducing FBx (SBx, n = 2309), we performed a propensity score matching on a 1:1 ratio, adjusting for age, number of positive biopsy cores, and initial PSA (iPSA). Results: A total of 492 patients undergoing FBx or SBx was matched. In total, 55% of patients diagnosed with GS 6 by FBx were upgraded to clinically significant PCa (defined as GS ≥ 7a) after RP, compared to 52% of patients diagnosed by SBx (*p* = 0.76). A time delay between FBx and RP was identified as the only correlate associated with upgrading. A total of 5.9% of all FBx patients and 6.1% of all SBx patients would have been eligible for AS (*p* > 0.99) but decided to undergo RP. The positive predictive value of AS eligibility (diagnosis of low-risk PCa after biopsy and after RP) was 17% for FBx and 6.7% for SBx (*p* = 0.39). Conclusions: In this study, we show, in a real-world setting, that introducing FBx did not lead to significant change in ratio of adverse pathology for low-risk PCa patients after RP compared to SBx.

## 1. Introduction

Treatment recommendations for prostate cancer (PCa) depend on risk stratification by predominantly histopathological and clinical findings [1]. Tissue samples were traditionally obtained by a systematic randomized transrectal ultrasound-guided prostate biopsy (SBx). In recent years, multiparametric magnetic resonance imaging (mpMRI) of the prostate has proved to significantly enhance the detection rate of clinically significant PCa (defined as histopathological grading ≥ Gleason score (GS) 7a) [2]. The likelihood of clinically significant PCa is assessed by the Prostate Imaging Reporting and Data System (PI-RADS) on a Likert scale of 1–5, with 5 expressing a very high likelihood of clinically significant PCa [3]. Randomized controlled studies showed superior detection rates of clinically significant PCa by mpMRI–ultrasound fusion-guided biopsy of the prostate (FBx) compared to SBx [4]. Therefore, current guidelines recommend a mpMRI prior to biopsy of the prostate [1].

Men with low-risk PCa following conservative management have a minimal risk of dying from PCa [5]. Low-risk PCa is defined as GS < 7, a serum prostate-specific antigen value (PSA) of less than 10 ng/mL and a clinical tumor involvement of half of one lobe (clinical stage T2a according to the International Society of Uropathologists TNM-classification) or less [1]. Therefore, for patients with low-risk PCa and a life expectancy of more than 10 years, active surveillance (AS) is increasingly becoming the preferred approach [6,7].

Before opting for AS, a reliable risk stratification is crucial [1,8]. However, it has been shown that the upgrading of PCa initially triaged as low-risk PCa to clinically significant PCa is a common finding when examining the whole prostate after radical prostatectomy (RP) [9,10,11,12,13]. Furthermore, studies showed that mpMRI detects less than half of all and less than two-thirds of clinically significant PCa foci, respectively [14]. Studies comparing upgrading rates of SBx and FBx after RP show controversial results [9,11,12,13,15]. Different experience levels of the involved specialties may bias previous analyses. In detail, it has been shown that there is a significant learning curve for the assessment of the PI-RADS score by radiologists [16], for the technique of FBx by urologists [17,18], and for the examination of the prostate biopsy samples by pathologists [19].

High-volume studies have postulated that patients who underwent FBx prior to enrolment in AS were less likely to experience reclassification during follow-up in comparison to patients who underwent SBx, suggesting a benefit of FBx in risk assessment [20]. In a large prospective cohort of 1818 patients under AS at Johns Hopkins University, Tosoian et al. showed that patients who underwent FBx prior to enrolment to AS were less likely to undergo upgrading on repeated biopsy compared to patients who underwent SBx prior to AS [19]. In the outpatient clinic of our department, we have been performing FBx based on mpMRI by external radiology centers for seven years. These centers comprise university clinics as well as private practices with a heterogeneous experience in assessing mpMRI. Before FBx at our outpatient clinic, mpMRI did not get reviewed by in-house radiologists.

The aim of this study was to present a realistic everyday life approach with a heterogeneous mix of different experience levels of the involved professions. We conducted a propensity-score matching to address the question if there are significant differences of FBx compared to SBx in up- and downgrading rates after RP and the consecutive positive predictive value of AS eligibility.

## 2. Subjects and Methods

### 2.1. MpMRI/Ultrasound Fusion-Guided Transrectal Prostate Biopsy (FBx)

**Figure 1:**
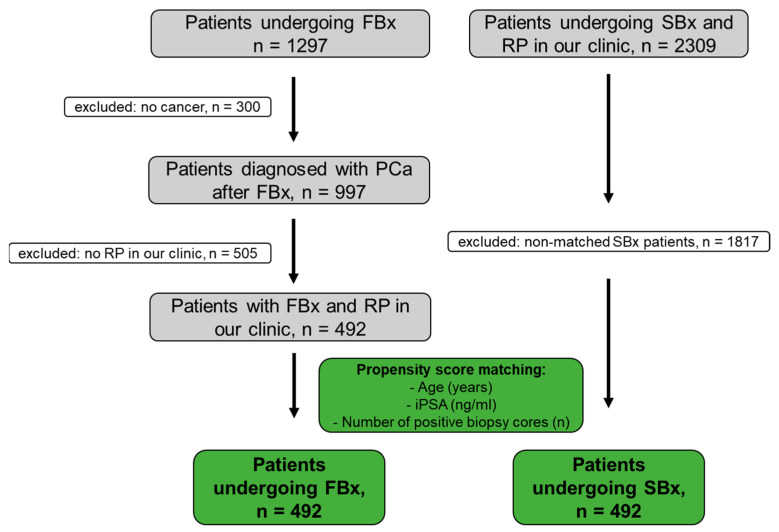
Cohort acquisition of the mpMRI–ultrasound fusion-guided prostate biopsy (FBx) and the standardized biopsy (SBx) for propensity score matching. PCa: prostate cancer, RP: radical prostatectomy.

The study design is summarized in Figure 1. Between March 2015 and March 2022, 1297 patients underwent FBx at the Department of Urology of the Ludwig-Maximilians-University of Munich, Germany. Patients were either referred by their office urologist or by the outpatient clinic of the Department of Urology of the Ludwig-Maximilians-University of Munich, Germany. A mpMRI for FBx was performed by the patient’s local radiologists. A secondary review of the mpMRI was not performed before FBx. A mpMRI assessed by 111 radiology centers was included in this study. FBx was performed by 14 urologists from our department with different levels of experience. FBx of one to three mpMRI lesions was performed and a mean of three cores per target was obtained. Fusion of mpMRI and ultrasound was performed using plane-wise fusion. The axial T2-weighted MRI sequence was used for image fusion. The targeted biopsy was performed by a transrectal ultrasound-guided biopsy system (Epiq7, Philips Percunav^®^, Philips Medical Systems, Bothell, WA, USA). Following current guidelines, a concurrent SBx was performed after FBx [1]. Therefore, 6 cores from each side of the prostate were obtained. 

Findings were reported based on the STROBE statement for cohort studies [21]. PCa was diagnosed in 997 of the 1297 patients (77%). Subsequently, 492/997 patients (49%) underwent radical prostatectomy in our clinic (open retropubic or robotically assisted). Data completeness was 100% (492/492 patients) for age, number of positive cores, number of cores taken, initial PSA, target positivity, number of targets, highest PI-RADS score, and time between FBx and RP. For highest infiltration at FBx, prior biopsy status, prostate volume, PSA density, and result of the digital rectal examination (DRE), the data completeness was 99.8% (491/492), 84% (412/492), 81% (397/492), 81% (397/492), and 47% (233/492), respectively. This study was approved by the local ethics committee (#22-0318).

### 2.2. Systematic Randomized Transrectal Ultrasound-Guided Biopsy of the Prostate (SBx)

A total of 2309 patients underwent SBx (12 bioptic cores) and consecutive RP at our department between July 2004 and September 2022 and was retrospectively identified and considered for analysis.

### 2.3. Propensity Score Matching and Statistical Analysis

All patients who underwent FBx were matched with those who underwent SBx on a 1:1 ratio through an optimal propensity score matching. The two groups were adjusted for age, number of positive biopsy cores, and iPSA. Covariate balance was evaluated with Love plots, and an absolute standardized mean difference below 0.1 indicated adequate matching balance. Baseline variables were calculated as mean and standard deviation (SD) or as frequencies with percentages. All parameters were assessed for normality with histograms and with the Shapiro–Wilk test, and the corresponding comparisons were performed using the χ^2^ or the two-sample *t*-test. The χ^2^ test was also performed to compare the up- and downgrading rates between FBx and SBx. We undertook a multivariable logistic regression analysis to identify potential correlates leading to upgrading of the Gleason score (GS) after radical prostatectomy. Based on clinical relevance, age, the proportion of positive prostate biopsy cores, the degree of infiltration of prostate cancer in the positive prostate biopsy cores, the initial PSA, the PI-RADS score, and the time elapsed between prostate biopsy and radical prostatectomy were selected as potential estimates. For all associations, odds ratios (ORs) and 95% confidence intervals (CIs) were estimated. All statistical tests were performed with the R statistical program (version 3.6.3) and the DATAtab Statistics Calculator (DATAtab e.U., Graz, Austria). *p*-values lower than 0.05 were considered statistically significant (two-sided).

## 3. Results

A total of 492 patients underwent FBx and consecutive RP in our department. Patient clinicopathological characteristics are displayed in Table 1. PCa was detected in the mpMRI target in 82% of patients. Patients undergoing FBx and subsequent RP (n = 492) were matched in a 1:1 ratio with those undergoing SBx and subsequent RP. Matching was adjusted for age, iPSA, and number of positive biopsy cores. Patient characteristics of the matched patients are depicted in Table 2. The mean patient age was 67 years ± 8 years for FBx patients and 67 years ± 8 years for SBx patients (*p* = 0.74), respectively. The mean number of positive biopsy cores was 5 ± 3 and 5 ± 3 for FBx and SBx, respectively (*p* = 0.59). The mean iPSA was 11.9 ng/mL ± 19.8 ng/mL for FBx and 12.4 ng/mL ± 18.9 ng/mL for SBx (*p* = 0.67). Propensity matching led to evenly distributed groups regarding key prognostic parameters. After propensity score matching, the rate of GS 6 after biopsy was significantly higher for FBx than for SBx (27% vs. 18%; *p* = 0.001). However, there was no difference in AS eligibility rate after FBx or SBx (5.9% vs. 6.1%, *p* > 0.99) and there was no significant difference (*p* = 0.35) in the rates for downgrading (FBx 19% vs. SBx 18%), concordance (FBx 52% vs. SBx 56%) and upgrading (FBx 29% vs. SBx 26%) after subsequent RP.

Patient characteristics of the GS 6 patients of the matched cohorts are depicted in Table 3. The mean patient age was 66 ± 8 years for FBx and 64 ± 8 years for SBx (*p* = 0.10). There was no significant difference between the mean number of positive biopsy cores (4 ± 2 for FBx and 3 ± 2 for SBx, *p* = 0.11) and iPSA (9.7 ng/mL ± 9.8 ng/mL for FBx and 7.9 ng/mL ± 3.9 ng/mL for SBx, *p* = 0.057), proving evenly distributed groups. For GS 6, there was no significant difference (*p* = 0.76) in the rates for concordance (FBx 45% vs. SBx 48%) and upgrading (FBx 55% vs. SBx 52%) after subsequent RP.

Patient characteristics of the patients assessed as eligible for active surveillance after biopsy are depicted in Table 4. For the AS-eligible patients of the matched cohorts, the mean patient age was 65 ± 8 years for FBx and 64 ± 8 years for SBx (*p* = 0.78). Moreover, there was no significant difference between the mean number of positive biopsy cores (2 ± 1 for FBx and 1 ± 1 for SBx, *p* =0.093) and iPSA (6.0 ng/mL ± 1.8 ng/mL for FBx and 6.5 ng/mL ± 1.8 ng/mL for SBx, *p* = 0.31), proving evenly distributed groups. For patients eligible for AS, there was also no significant difference (*p* > 0.99) in the rates for concordance (FBx 45% vs. SBx 47%) and upgrading (FBx 55% vs. SBx 53%) of GS. The positive predictive value for AS eligibility was 17% for FBx compared to 6.7% for SBx (*p* = 0.39).

### 3.1. Subgroup Analysis of Up- and Downgrading Rates Regarding Gleason Scores

**Figure 2:**
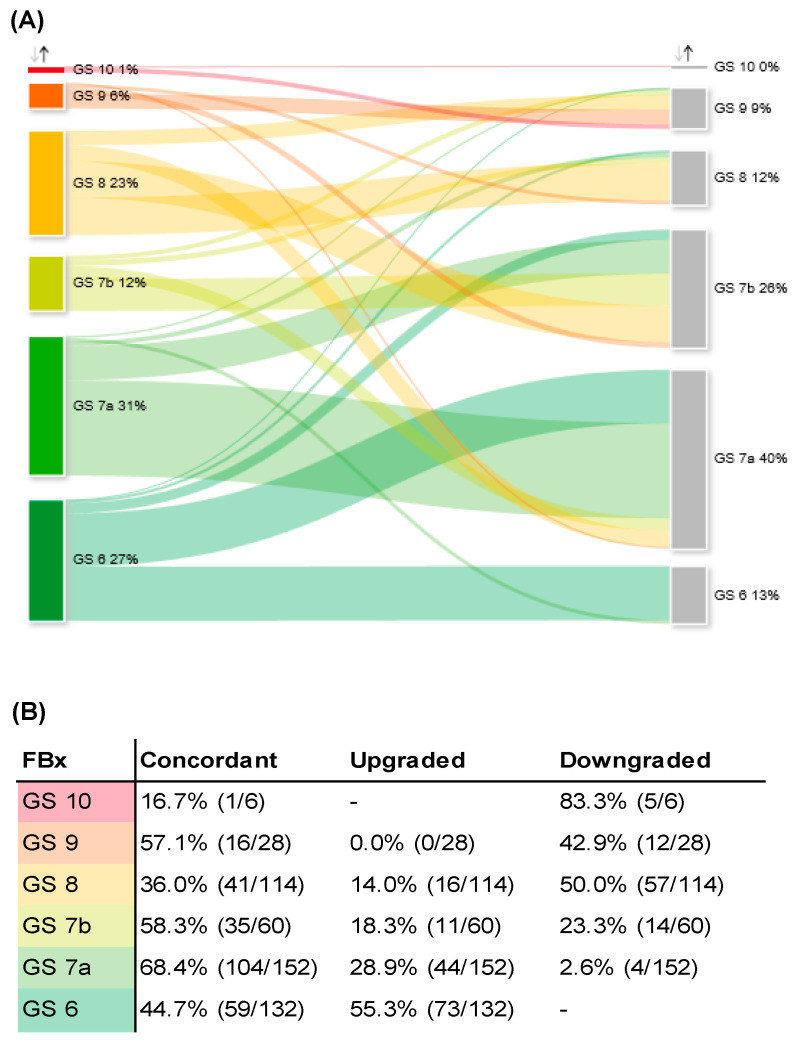

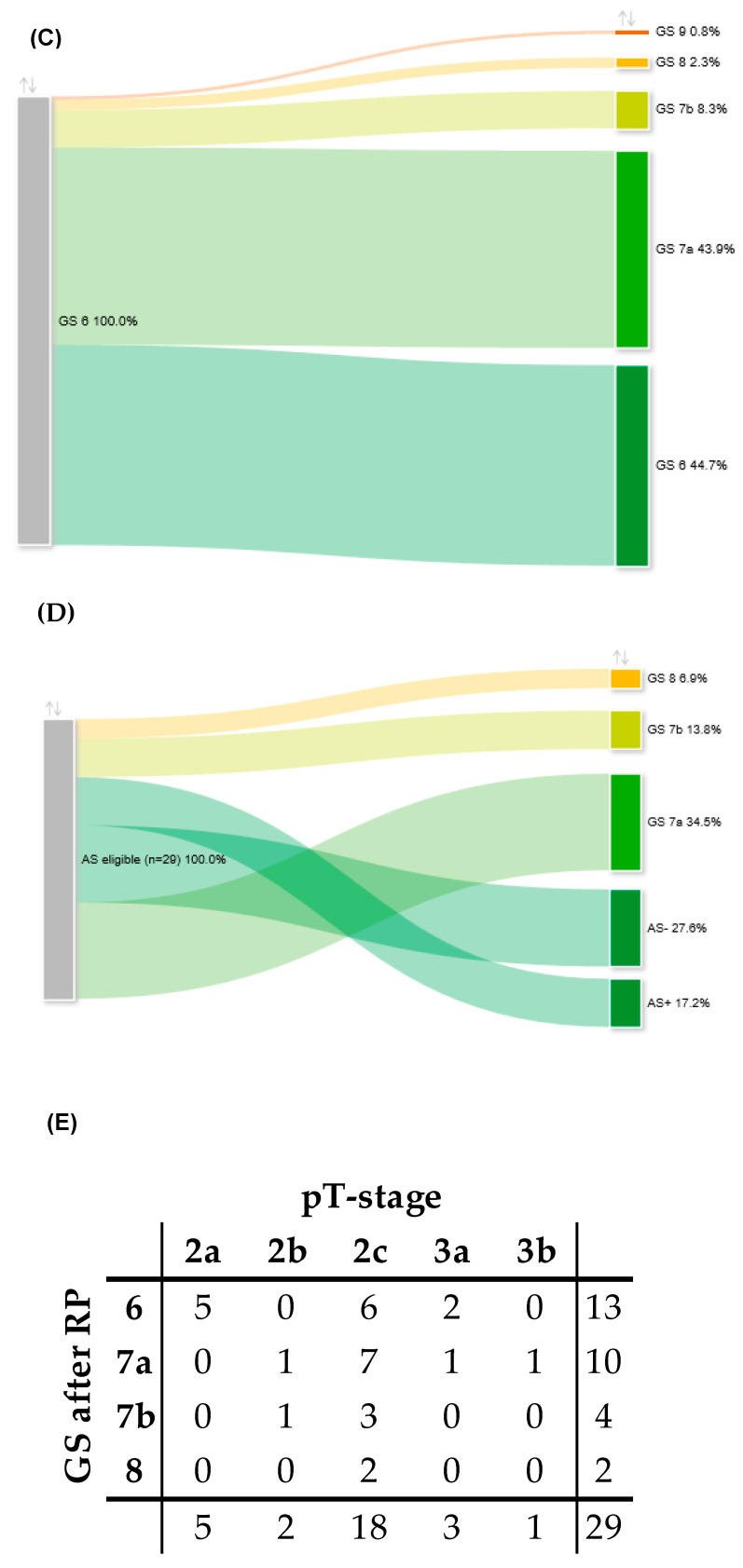
(**A**) Sankey diagram of change in GS comparing FBx specimen (left column) and RP specimen (right column). (**B**) Concordant, up- and downgrading rates of different GS after FBx and consecutive RP as depicted in Figure 2A. (**C**) Sankey diagram of adverse pathology of GS 6 PCa after FBx (left column) and consecutive RP (right column). (**D**) Sankey diagram of upgrading and -staging of patients assessed as eligible for AS after FBx (left column) and consecutive RP (right column). (**E**) Cross chart of histopathological characteristics of RP specimen of all patients who were assessed as eligible for AS after FBx. GS: Gleason score, FBx: mpMRI fusion-guided biopsy of the prostate, RP: radical prostatectomy, PCa: prostate cancer, AS: active surveillance, AS+: local stage pT2a (pathological tumor stage according to the International Society of Uropathologists TNM-classification) and GS 6, AS−: local stage > pT2a and GS 6.

Detailed analysis of GS grading of FBx and RP specimens is depicted in Figure 2A,B. Overall, 30.9% and 12.2% of patients undergoing RP were diagnosed with GS 7a and 7b by FBx, respectively. After RP, 39.6% and 26.2% were graded as GS 7a and 7b, respectively. GS 7a had the highest concordance rate of GS grading after RP (68.4%), whereas GS 6 had the highest upgrading rate (55.3%).

### 3.2. Adverse Pathology of Gleason Score 6 Prostate Cancer Diagnosed by FBx

A detailed analysis of adverse pathology of GS 6 is depicted in Figure 2C. Concerning GS 6 detected by FBx, 44.7% of the respective RP specimens were concordantly graded as GS 6. A total of 55.3% was upgraded to adverse pathology. We did not observe a significant difference in risk of adverse pathology of GS 6 for FBx compared to SBx (FBx 55%, SBx 52%, *p* = 0.76).

### 3.3. Upgrading and -Staging of Patients Eligible for Active Surveillance

Based on the EAU criteria for active surveillance (AS), we further analyzed a subgroup of patients of the GS 6 cohort, who would have been eligible for AS but chose to undergo RP at our department. A total of 29/132 (22%) and 217/635 (34%) of all patients with GS 6 would have been eligible for AS but decided to undergo RP after FBx and SBx, respectively. In total, 29/492 (5.9%) patients would have been eligible for AS determined by FBx. After RP, 5/29 patients (17.2%) showed concordant GS 6 and local stage pT2a and, thus, would have still met the EAU criteria for AS. In 8/29 patients (27.6%), the RP specimen showed concordant GS 6 but a local stage of ≥pT2b, excluding these patients for AS postoperatively. In 10/29 patients (34.5%), an upgrading to GS 7a was observed, and in 4/29 patients (13.8%), an upgrading to GS 7b was observed. A total of 2/29 patients (6.9%) showed an upgrading to GS 8 in the RP specimen. In 4/29 patients (13.8%), the tumor showed extracapsular extension in the RP specimen (≥pT3a). Upgrading and -staging of patients that would have been eligible for AS after FBx is depicted in Figure 2D,E. In the multivariable analysis, we could not identify a potential correlate leading to upgrading in the AS cohort (supplementary Table S1). Of the matched patients that were assessed as eligible for AS after SBx, 28/30 patients (93.3%) experienced upgrading, upstaging, or both after RP. To sum up, after RP, the positive predictive value for AS eligibility was 17.2% for FBX and 6.7% for SBx (*p* = 0.39).

### 3.4. Univariate and Multivariable Logistic Regression Analyses: Up- and Downgrading after RP

To identify potential correlates leading to upgrading after RP, we performed univariate and multivariable logistic regression analyses adjusted for age, the number of positive prostate biopsy cores, the degree of tumor infiltration per biopsy core, PSA value, PI-RADS score, and the time delay between FBx and RP. The univariate and multivariable logistic regression analyses are depicted in Table 5A,B. In univariate analysis, time delay between FBx and RP was significantly longer in patients who were upgraded after RP compared to patients who received no upgrading after RP (84.7 months vs. 56.9 months, *p* = 0.031). Furthermore, in multivariable analysis, time delay between FBx and RP was identified as a potential correlate leading to upgrading (OR 1.01, 95% CI 1.01–1.01, *p* = 0.001). On further analysis, a subgroup of 132 patients diagnosed with GS 6 by FBx was evaluated by univariate and multivariable logistic regression analyses to identify potential correlates of adverse pathology after RP. No significant correlate was identified to predict adverse pathology after RP for patients diagnosed with GS 6 by FBx (supplementary Table S3). Longitudinal ratio of upgrading rates over the evaluated seven years showed no statistical change over time (*p* = 0.53; supplementary Figure S1). The results of univariate and multivariable logistic regression analyses concerning downgrading after RP are depicted in Table 5C,D. Here, the percentage of positive FBx cores was identified as a potential correlate leading to downgrading in both univariate (*p* < 0.001) and multivariable logistic regression analyses (OR 6.14, 95% CI 1.99–19.1, *p* < 0.001).

## 4. Discussion

The risk of false diagnosis by prostate biopsy is a major element of uncertainty for treatment decision. However, a rational risk stratification is crucial, particularly for patients deciding to undergo AS [1]. We retrospectively analyzed the reliability of FBx and SBx in the diagnosis of insignificant PCa in two large contemporary cohorts with propensity score matching. Our findings indicate that there is no significant difference between the total up- and downgrading rates of FBx compared to SBx after RP. Most patients diagnosed with GS 6 were upgraded to clinically significant PCa after RP, irrespective of the approach of prostate biopsy. Most patients assessed as eligible for AS by FBx and SBx would not have met the inclusion criteria after RP. In the univariate and multivariable analysis, the time between FBx and RP was the only correlate leading to upgrading of GS. Although not statistically significant, the positive predictive value for AS eligibility determined by FBx was higher compared to SBx. In earlier studies, FBx has undoubtedly proven diagnostic benefits in detection of clinically significant PCa in comparison to the conventional SBx approach [22]. Data on the diagnostic value of FBx in risk stratification for AS eligibility are sparse. Furthermore, earlier reports comparing upgrading rates between FBx and SBx might be biased by different levels of experience of involved radiologists, urologists, and pathologists [9,11,12,13,15]. In our analysis, we present a realistic everyday life approach of FBx with a heterogeneous mix of the included specialties. To our knowledge, our study comprises the largest evaluated single-center cohort on this issue.

In a national, multicenter observational study from the United Kingdom, including 17,598 PCa patients, the concordance, upgrading, and downgrading rates between biopsy and RP specimen were reported to be 59%, 26%, and 16%, respectively [23]. This is in line with our results, with concordance rates of 52% and 56%, upgrading rates of 29% and 26%, and downgrading rates of 19% and 18% for FBx and SBx, respectively. Our analysis observed a trend toward GS 7a and GS 7b in the RP specimen. A “regression to the mean” has been observed before and it was argued that the examination of the whole prostate leads to a higher prevalence of mixed Gleason 3 and 4 patterns [24]. Whether patients undergoing FBx have different upgrading rates after RP compared to patients undergoing SBx remains controversially discussed [9,11,12,13,15]. In the present study, there was no significant difference in overall upgrading rates between FBx and SBx (29% vs. 26%, *p* = 0.35). Furthermore, we could not find any significant difference in the risk for adverse pathology of GS 6 between FBx and SBx (45% vs. 48%, *p* = 0.76).

In regression analyses of potential correlates, we found a significant association between upgrading of PC and the time delay between FBx and RP. Ginsburg et al. evaluated the association between delay of biopsy and subsequent RP and upgrading in 128,062 men [25]. In contrast to our study, they could not find a significant difference between patients treated with immediate RP and those with a delay of up to 12 months [25]. However, they exclusively included patients with intermediate and high-risk PCa in their analyses [25]. Not including low-risk patients might constitute a potential bias, as this patient group represented the highest upgrading rate among all groups in our study. This might explain the discrepancy to our results. Bullock et al. analyzed 17,598 PCa patients undergoing RP after FBx or SBx and reported that the low-risk cohort had the highest upgrading rate (55.7%) of all risk groups [23]. In our analysis, age or PSA level were not significantly associated with adverse pathology, in contrast to previous studies [23,26]. In the study mentioned above, Bullock et al. reported significantly higher PSA values of those patients who were upgraded compared to those who were not (10.8 ng/mL vs. 9.81 ng/mL, *p*< 0.001). Björnebo et al. analyzed a cohort of 6021 patients with low-risk PCa that initially underwent AS. They reported that PSA (OR 1.22, 95% CI 1.07–1.41) and age (OR 1.09, 95% CI 1.02–1.18) were significantly associated with upgrading after RP [26]. In our analysis, we could not confirm these results. Considering the learning curves for assessment of mpMRI by radiologists [16], for the technique of FBx by urologists [17,18], and for the examination of the prostate biopsy by pathologists [19], one could have expected an overall improvement in the concordance of GS over time. However, there was no change in the upgrading rate over the 7 years of our analysis.

Most patients of our study who were initially assessed as eligible for AS did not meet the inclusion criteria for AS after RP. Tosoian et al. monitored 1818 men on AS for 5 years and reported that not undergoing mpMRI prior to enrollment was associated with an increased risk for reclassification during follow-up (hazard ratio 1.46; 95% CI 1.07–2.63; *p* = 0.04) [20]. In our study, although not statistically significant, the positive predictive value for AS eligibility was higher for FBx compared to SBx (17.2% vs. 6.7%; *p* = 0.39). The percentage of low-risk PCa patients opting for AS is constantly increasing [6,7]. Likewise, we observed a substantial decrease in AS-eligible patients undergoing RP at our department over the past years. In an analysis of patients undergoing RP in our department between 2004 and 2007, 36.3% (308/849 patients) met the criteria for AS but decided to undergo RP [10]. In the present analysis of the years 2015–2022, only 5.9% (29/492 patients) met the criteria for AS but decided to undergo RP. This might represent recent changes in risk-adapted therapy of PC. Suitably, data from our clinic previously described a significant stage migration of patients presenting for RP throughout the last decade toward more aggressive and locally advanced PCa [27].

Recently, it has been pointed out that GS 6 PCa should rather be considered a precancerous state treated best by close monitoring [28]. However, our study highlights the risk of false-negative diagnosis when enrolling patients in AS. For upgraded GS 6 patients, it has been shown that the rate of biochemical recurrence is higher compared to those with concordant histopathology after RP [24]. In line with previous reports, we report that a substantial proportion of men eligible for AS display aggressive tumor features after RP [29,30]. Of all patients diagnosed as AS eligible by FBx in our study, 13.8% showed extracapsular tumor extension after RP. This is in line with previously reported results [29,30]. In a study by Porten et al., who analyzed 377 patients undergoing AS, 34% were found to experience upgrading over the course of AS [31]. The majority of those experiencing an upgrading (81%) did so by their second repeat biopsy [31]. The authors pointed out that an early upgrading was most likely due to an initial sampling error, whereas later upgrading may reflect tumor dedifferentiation [31]. In the future, we might experience great advances in diagnostics and risk stratification for prostate cancer. This might be due to novel imaging modalities or through the potential power of artificial intelligence techniques. Radiomics in prostate cancer might be one of those techniques [32]. For now, we conclude that patients assessed as AS eligible by FBx or SBx should be informed about the low positive predictive values for AS eligibility.

## 5. Limitations

It should be noted that there are several limitations of the present study. First and foremost, it is a single-center retrospective analysis of a prospectively maintained database. Still, 111 different radiology centers and 14 urologists performing FBx were involved reflecting real-world data in that manner. Moreover, only 49% (492/997) of all patients diagnosed with PCa by FBx underwent RP in our department, which might have led to a selection bias, especially in GS 6 and AS-eligible patients. Notably, the trend to concentrate high-risk patients in high-volume centers may be an additional source of a selection bias with a higher proportion of this risk group in our analysis.

## 6. Conclusions

Our findings indicate that the introduction of FBx did not lead to a significant change in the ratio of adverse pathology of GS 6 PCa compared to the SBx. Even though we provide evidence that the positive predictive value for AS eligibility might be higher for FBx than for SBx, our study was underpowered to demonstrate any statistical significance between the two approaches. We present real-world data from a plethora of heterogeneously experienced radiologists, urologists, and pathologists. We conclude that patients with low-risk PCa opting for AS should be informed about the significant risk of being undergraded, understaged, or both. However, only a prospective trial may disclose the real impact of upgrading or -staging on survival.

## Figures and Tables

**Table 1:** Patient characteristics of the 492 included FBx patients. Data are shown as mean and standard deviation (SD) or as ratio in percent. FBx: mpMRI–ultrasound fusion-guided prostate biopsy, RP: radical prostatectomy, iPSA: prostate-specific antigen at FBx, DRE: digital rectal examination.

Patient Characteristics	All FBx Patients (n = 492)
Age (years)	67.0 ± 8.0
Number of positive biopsy cores (n)	5 ± 3
Number of biopsy cores obtained (n)	13 ± 1
Highest infiltration of biopsy cores (%)	49 ± 24
iPSA (ng/mL)	11.9 ± 19.8
Detection rate of PCa (%)	82 (401/492)
Prior biopsy (%)	28 (117/412)
Number of mpMRI targets per patient (n)	1 ± 0.5
Highest PI-RADS score	4 ± 0.7
Prostate volume (mL)	50.5 ± 29.8
PSA Density (ng/mL/ccm)	0.26 ± 0.31
Positive DRE (%)	54 (126/233)
Time between FBx and RP (days)	65.0 ± 101.4

**Table 2:** Baseline characteristics of patients undergoing FBx versus SBx. Values are presented as mean ± standard deviation or n (%). The *t*-test was performed for comparisons between continuous variables and the chi-squared test between categorical variables. AS: active surveillance, GS: Gleason score, iPSA: prostate-specific antigen at FBx, RP: radical prostatectomy.

Propensity Score Matching	Overall, n = 984	SBx, n = 492	FBx, n = 492	*p*-Value
Gleason score after RP				0.35
Downgrading from biopsy	181 (18%)	89 (18%)	92 (19%)	
Concordance with biopsy	533 (54%)	277 (56%)	256 (52%)	
Upgrading from biopsy	270 (27%)	126 (26%)	144 (29%)	
Age [years]	67.1 ± 7.8	67.2 ± 7.7	67.0 ± 8.0	0.74
Number of positive biopsy cores [n]	5.3 ± 3.1	5.3 ± 3.2	5.2 ± 3.0	0.59
iPSA [ng/mL]	12.1 ± 19.3	12.4 ± 18.9	11.9 ± 19.8	0.67
GS 6 after biopsy	220 (22%)	88 (18%)	132 (27%)	0.001
AS eligible after biopsy	59 (6.0%)	30 (6.1%)	29 (5.9%)	>0.99

**Table 3:** Baseline characteristics of patients undergoing FBx versus SBx with a Gleason score 6 after biopsy. Values are presented as mean ± standard deviation or n (%). The *t*-test was performed for comparisons between continuous variables and the chi-squared test between categorical variables. iPSA: prostate-specific antigen at FBx, RP: radical prostatectomy.

Gleason Score 6 after Biopsy	Overall, n = 220	SBx, n = 88	FBx, n = 132	*p*-Value
Gleason score after RP				0.76
Concordance with biopsy	101 (46%)	42 (48%)	59 (45%)	
Upgrading from biopsy	119 (54%)	46 (52%)	73 (55%)	
Age [years]	65.3 ± 7.6	64.3 ± 7.7	66.0 ± 7.5	0.10
Number of positive biopsy cores [n]	3.6 ± 2.3	3.3 ± 2.2	3.8 ± 2.4	0.11
iPSA [ng/mL]	9.0 ± 8.0	7.9 ± 3.9	9.7 ± 9.8	0.057

**Table 4:** Baseline characteristics of patients undergoing FBx versus SBx assessed as eligible for active surveillance after biopsy. Values are presented as mean ± standard deviation or n (%). The *t*-test was performed for comparison between continuous variables and the chi-squared test between categorical variables. AS: active surveillance, iPSA: prostate-specific antigen at FBx, RP: radical prostatectomy.

Active Surveillance Eligible after Biopsy	Overall, n = 59	SBx, n = 30	FBx, n = 29	*p*-Value
Gleason score after RP				>0.99
Concordance with biopsy	27 (46%)	14 (47%)	13 (45%)	
Upgrading from biopsy	32 (54%)	16 (53%)	16 (55%)	
Age [years]	64.2 ± 7.6	64.0 ± 7.8	64.5 ± 7.5	0.78
Number of positive biopsy cores [n]	1.4 ± 0.5	1.3 ± 0.5	1.5 ± 0.5	0.093
iPSA [ng/mL]	6.3 ± 1.8	6.5 ± 1.8	6.0 ± 1.8	0.31
Positive predictive value for AS eligibility (diagnosis of low risk PCa after biopsy and after RP)	7 (12%)	2 (6.7%)	5 (17%)	0.39

**Table 5:** Baseline characteristics, as well as univariate and multivariable logistic regression analyses, of all upgraded (**A**,**B**) and downgraded (**C**,**D**) FBx patients. Continuous variables are presented as mean and standard deviation. Categorical variables are presented in absolute numbers and relative numbers in percent. FBx: mpMRI fusion-guided biopsy of the prostate, RP: radical prostatectomy, iPSA: prostate-specific antigen at the time of RP, PI-RADS: Prostate Imaging Reporting and Data System, OR: odds ratio, CI: confidence interval.

**(A)**									**(C)**								
**Baseline characteristics of all upgraded patients**									**Baseline characteristics of all downgraded patients**								
**Baseline Characteristics**				**Upgrading, n = 144**		**No upgrading, n = 348**		***p*** **-value**	**Baseline Characteristics**				**Downgrading, n = 92**	**No downgrading, n = 400**			***p*** **-value**
**Age (years)**				67.8 ± 7.2		66.7 ± 8.2		0.13	**Age (years)**				68.2 ± 7.9	66.8 ± 8.0			0.12
**Number of positive biopsy cores (n)**				5.3 ± 3.1		5.2 ± 2.9		0.58	**Number of positive biopsy cores (n)**				6.2 ± 3.0	5.0 ± 2.9			<0.001
**Number of biopsy cores obtained (n)**								0.80	**Number of biopsy cores obtained (n)**								0.10
**Highest infiltration of the obtained biopsy cores (%)**				50.3 ± 25.2		48.0 ± 23.6		0.36	**Highest infiltration of the obtained biopsy cores (%)**				50.7 ± 23.8	48.2 ± 24.1			0.38
**iPSA (ng/mL)**				14.1 ± 32.8		10.9 ± 10.4		0.26	**iPSA (ng/mL)**				12.2 ± 13.3	11.8 ± 21.0			0.79
**Prior biopsy (n)**				38 (30.9%)		79 (27.3%)		0.54	**Prior biopsy (n)**				23 (29.1%)	94 (28.2%)			0.99
**Highest PI-RADS score**								0.086	**Highest PI-RADS score**								0.75
3				19 (13.2%)		28 (8.0%)			3				9 (9.8%)	38 (9.5%)			
4				54 (37.5%)		161 (46.3%)			4				37 (40.2%)	178 (44.5%)			
5				71 (49.3%)		159 (45.7%)			5				46 (50.0%)	184 (46.0%)			
**Prostate volume (mL)**				50.2 ± 26.1		50.6 ± 31.1		0.91	**Prostate volume (mL)**				57.6 ± 36.1	48.8 ± 27.9			0.048
**Positive DRE**				28 (50.9%)		98 (55.1%)		0.70	**Positive DRE**				38 (65.5%)	88 (50.3%)			0.062
**Time between FBx and RP (days)**				84.7 ± 145.4		56.9 ± 75.0		0.031	**Time between FBx and RP (days)**				43.9 ± 34.7	69.9 ± 110.7			<0.001
**T Stage after RP**								0.012	**T Stage after RP**								0.059
2a				4 (2.8%)		20 (5.7%)			2a				2 (2.2%)	22 (5.5%)			
2b				4 (2.8%)		6 (1.7%)			2b				5 (5.4%)	5 (1.2%)			
2c				66 (45.8%)		208 (59.8%)			2c				47 (51.1%)	227 (56.8%)			
3a				43 (29.9%)		71 (20.4%)			3a				23 (25.0%)	91 (22.8%)			
3b				27 (18.8%)		43 (12.4%)			3b				15 (16.3%)	55 (13.8%)			
**(B)**								**(D)**									
**Baseline characteristics of all upgraded patients**								**Baseline characteristics of all downgraded patients**									
**Characteristic**	**Univariate**				**Multivariable**			**Characteristic**		**Univariate**			**Multivariable**				
	**OR**	**95% CI**	***p*** **-value**		**OR**	**95% CI**	***p*** **-value**			**OR**	**95% CI**	***p*** **-value**	**OR**		**95% CI**	***p*** **-value**	
**Age**	1.02	0.99, 1.04	0.15		1.02	0.99, 1.04	0.20	**Age**		1.02	0.99, 1.05	0.12	1.02		0.99, 1.05	0.20	
**Percent of positive biopsy cores**	1.12	0.47, 2.64	0.80		1.02	0.36, 2.78	>0.9	**Percent of positive biopsy cores**		5.78	2.19, 15.3	<0.001	6.14		1.99, 19.1	<0.001	
**Highest infiltration of the obtained biopsy cores**	1.00	1.00, 1.01	0.30		1.00	1.00, 1.01	0.30	**Highest infiltration of the obtained biopsy cores**		1.00	0.99, 1.01	0.40	0.99		0.98, 1.01	0.30	
**iPSA**	1.01	1.00, 1.02	0.20		1.01	1.00, 1.02	0.20	**iPSA**		1.00	0.99, 1.01	0.80	1.00		0.98, 1.01	0.80	
**Highest PI-RADS score**	0.96	0.72, 1.30	0.80		0.94	0.68, 1.30	0.70	**Highest PI-RADS score**		1.09	0.77, 1.56	0.60	0.88		0.60, 1.29	0.50	
**Time between FBx and RP**	1.01	1.01, 1.01	0.01		1.01	1.01, 1.01	0.01	**Time between FBx and RP**		0.99	0.99, 0.99	0.03	0.99		0.99, 1.00	0.07	

## Data Availability

The data presented in this study are available on request from the corresponding author. The data are not fully publicly available yet due to further ongoing studies, that are not published yet.

## Supplementary Materials

The following supporting information can be downloaded at: https://www.mdpi.com/article/10.3390/bioengineering10020247/s1, Figure S1: Upgrading rate of all FBx patients (mpMRI fusion-guided biopsy of the prostate) after radical prostatectomy over time; Table S1: Characteristics of the upgrading in the FBx AS cohort; Table S2: Upgrading and -staging of AS-eligible SBx patients; Table S3: Univariate and multivariable logistic regression analyses of upgraded GS 6 FBx patients.
