# Supplementary material for: Adverse Pathology after Radical Prostatectomy of Patients Eligible for Active Surveillance—A Summary 7 Years after Introducing mpMRI-Guided Biopsy in a Real-World Setting
Source: Bioengineering (Basel). 2023 Feb 13;10(2):247. doi: 10.3390/bioengineering10020247 (PMC9952076; doi:10.3390/bioengineering10020247)
Supplement: Supplementary file 1 [file bioengineering-10-00247-s001.zip › bioengineering-2190243-supplementary.pdf]

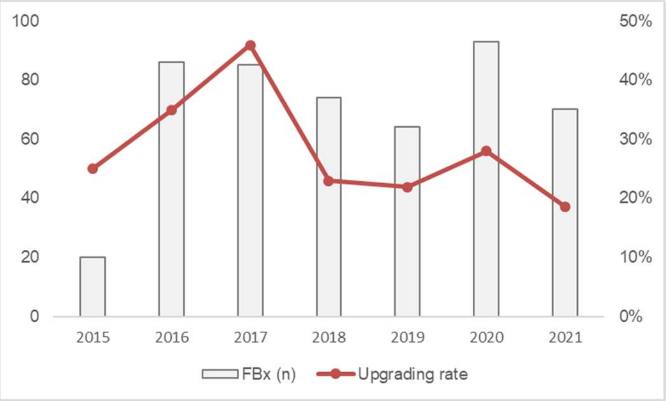

Supplementary Figure S1. Upgrading rate of all FBx patients (mp-MRI-fusion guided biopsy of the prostate) after radical prostatectomy over time. March 2015 – February 2016 vs. March 2021 – February 2022; *p* = 0.53.

Supplementary Table S1. Characteristics of the upgrading in the FBx AS cohort

Baseline Characteristic	Gleason score upgrading, n = 16	No Gleason score upgrading, n = 13	p-value
**Age (years)**	65.4 ± 8.3	63.5 ± 6.4	0.49
**Positive PB cores (n)**			0.36
1	6 (37.5%)	8 (61.5%)	
2	10 (62.5%)	5 (38.5%)	
**PB cores (n)**			0.093
**13**	12 (75.0%)	12 (92.3%)	
**14**	4 (25.0%)	0 (0.0%)	
**15**	0 (0.0%)	1 (7.7%)	
**Infiltration at PB (%)**	16.6 ± 14.3	10.6 ± 9.7	0.20
**iPSA (ng/ml)**	6.3 ± 1.7	5.7 ± 1.9	0.33
**Prior biopsy (n)**	5 (45.5%)	4 (36.4%)	>0.99
**Highest PI-RADS score**			0.59
**3**	3 (18.8%)	1 (7.7%)	
**4**	9 (56.2%)	7 (53.8%)	
**5**	4 (25.0%)	5 (38.5%)	
**Prostate volume (mL)**	42.3 ± 31.4	55.6 ± 25.5	0.31
**Positive DRE**	3 (60.0%)	1 (16.7%)	0.39

Baseline Characteristic	Gleason score upgrading, n = 16	No Gleason score upgrading, n = 13	p-value
**Time between PB and RP (days)**	186.4 ± 202.6	159.6 ± 240.2	0.75
**Mean operative time (min)**	106.5 ± 55.8	132.2 ± 63.2	0.26
**T Stage after RP**			0.12
**2a**	1 (6.2%)	5 (38.5%)	
**2b**	2 (12.5%)	0 (0.0%)	
**2c**	11 (68.8%)	6 (46.2%)	
**3a**	1 (6.2%)	2 (15.4%)	
**3b**	1 (6.2%)	0 (0.0%)	

Supplementary Table S2. Upgrading and staging of AS-eligible SBx patients.

Histopathology after SBx	Histopathology after RP	n =	%
GS 6, pT2a	GS 6 and **pT2a**	15	6.9
	GS 6 and **>pT2a**	111	51.2
	GS 7a	77	35.5
	GS 7b	10	4.6
	GS 8	2	0.9
	GS 9	2	0.9
	GS 10	0	0.0

Supplementary Table S3. Univariate and multivariable logistic regression analyses of upgraded GS 6 FBx patients (n = 132).

Characteristic	Univariate			Multivariable		
	OR	95% CI	p-value	OR	95% CI	p-value
**Age**	1.03	0.99, 1.08	0.20	1.01	0.96, 1.07	0.60
**Percent of positive PB cores**	11.80	1.49, 110	0.02	4.65	0.43, 58.2	0.20
**Highest infiltration oft he obtained biopsy cores**	1.02	1.01, 1.04	0.01	1.02	1.00, 1.03	0.10
**iPSA**	1.00	0.96, 1.03	0.80	1.00	0.96, 1.04	>0.9
**Highest PI-RADS score**	1.32	0.81, 2.18	0.30	1.28	0.76, 2.18	0.40
**Time between FBx and RP**	1.00	0.99, 1.01	0.80	1.00	0.99, 1.01	0.40
